# Impacts of Salinity Intrusion in Community Health: A Review of Experiences on Drinking Water Sodium from Coastal Areas of Bangladesh

**DOI:** 10.3390/healthcare7010050

**Published:** 2019-03-22

**Authors:** Mashura Shammi, Md. Mostafizur Rahman, Serene Ezra Bondad, Md. Bodrud-Doza

**Affiliations:** 1 Department of Environmental Sciences, Jahangirnagar University, Savar, Dhaka 1342, Bangladesh; 2 Graduate School of Environmental Science, Hokkaido University, Sapporo 060-0810, Japan; secbondad@gmail.com; 3 Climate Change Programme, BRAC, Dhaka 1212, Bangladesh; bodruddoza.env12@gmail.com

**Keywords:** salinity, drinking water sodium, hypertension, managed aquifer recharge, pond sand filter

## Abstract

Increasing salt intake has substantial negative impacts on human health and well-being. This article focused on the construction of Driver-Pressure-State-Impact-Response (DPSIR) framework for drinking water sodium (DWS) followed by a review on the published studies regarding salinity intrusion, DWS, and their effects on health perspectives in Bangladesh. Saline water is an important factor for hypertension or high blood pressure in the coastal areas. DWS can also lead women, especially pregnant women, to an increased risk of (pre)eclampsia, hypertension, as well as infant mortality. Several interventions, such as rainwater harvesting, pond sand filter (PSF) system, managed aquifer recharge (MAR), and pilot scale solar-powered desalination plants, such as reverse osmosis (RO), were reviewed on the context of their effectiveness in controlling drinking water sodium. Although rainwater consumption has the positive impact of low or no sodium intake, it still possesses negative impacts from not having vital minerals. A steady increment in sodium concentration through the span of the dry season was observed in MAR. It is, subsequently, important to increase awareness on DWS intake by providing and adopting correct technological interventions and training communities on the maintenance of the adaptive measures.

## 1. Introduction

Seawater intrusion is a pressing issue in coastal aquifers worldwide. Surface water resources, like rivers and canals, are severely affected by the intrusion of saline water [1,2]. In the mega-delta coastal areas of Vietnam, Bangladesh, and India, surface and near-surface drinking water are most susceptible to contamination by saline water intrusion, putting more than 25 million people at jeopardy of drinking saline water. Climate change is liable for intensifying this problem, which also has adverse health consequences, such as greater prevalence of hypertension and cardiovascular diseases [3]. The cumulative effects of salinity, arsenic contamination, and drought pose threats to the water quality and security, as well as health of coastal communities in Bangladesh [2]. Wherein, contamination of water occurs by varying degrees of salinity from rising sea levels, cyclone and storm surges, and upstream withdrawal of freshwater [4].

High salt intake is a major risk factor for increased blood pressure. Approximately 20 million people in Bangladesh are at high risk of hypertension due to the intrusion of saline water caused by climate change [5]. The physical geography of the coastal region of Bangladesh is more diverse and dynamic than usually predicted. Failure to acknowledge this has driven inaccurate assumptions on the conceivable impacts of rising ocean levels on Bangladesh due to global warming [6]. Most of Bangladesh’s coastal towns are located on the banks of low tidal areas at an average elevation of 1.0–1.5 m from the sea level [7]. The southern portion of coastal Bangladesh covers about 32% of the entire zone of the country [8] and is significantly susceptible to the effects of temperature variations due to climate change [9]. Water salinity is a consistent peril to numerous districts of southern Bangladesh. For instance, Batiaghata Upazila, located within the southwestern Khulna District of Bangladesh, is a frequent saline-affected area, in which agricultural activities mainly depend on precipitation [10]. An earlier study on groundwater in southwestern Bangladesh established that the zone was prevailing in NaCl category of brackish waters [11] because of the inductive influence of seawater and hydrogeochemical processes of the Bengal delta on the groundwater aquifer [12]. Anthropogenic activities such as upstream freshwater removal, and biophysical factors such as cyclones that originate outside the topographical frontier of the coastal Bangladesh, contribute to the cumulative increase in salinization in the southwestern region [13]. The average values of total dissolved solids (TDS), electrical conductivity (EC), and chloride concentration (Cl^−^) were found to be 4044.12 mg/L, 7186.7 mS/cm, and 3143.6 mg/L, respectively in Shyamnagar, and 2313.60 mg/L, 4390.3 mS/cm, and 1402.1 mg/L, respectively in Tala Upazila of Satkhira district, one of the severely affected areas of salinity in Bangladesh. Local people are aware of the safe water scarcity in the mentioned areas and nearly all of them perceive that salinity is the main reason behind it [14].

In addition, millions of people on the coastal regions of Southeast Asia experience cumulative sodium concentrations in their sources of potable water, which is partly influenced by climate change [15]. The long-term population health effects of consumption of substantial amounts of sodium through drinking water remain unknown [15]. About 20 percent of adults and 40–65 percent of elderly people in Bangladesh suffer from hypertension, which is an increasingly important medical and public health problem [16]. It has been proven that increased dietary sodium ingestion contributes to the risk of hypertension, but whether sodium ingestion through potable water could have parallel impacts on human health remains to be investigated [15]. A recently published study in Nature (January 2019) showed that Na^+^ consumption has immunological effects on skin tissue, intestinal microbiology, and other organs, as well as cardiovascular disease, inflammation, infection, and autoimmunity [17]. Drinking water sodium (DWS) is a critical source of daily sodium intake and a hypertension hazard to populations in salinity affected zones. In accordance with the World Health Organization (WHO) and the Food and Agriculture Organization (FAO) (2002) joint expert consultation, the prescribed nutritional sodium ingestion is 2 g/day (<85 mmol/day) [18]. However, DWS intake has exceeded in many parts of coastal Bangladesh due to salinity intrusion, climate change, storm surges, etc., in the soil and in the existing water resources. In line with the upsurge in salinization, swift courses of activities are required in the affected zones. Since managed aquifer recharge (MAR) has varying outcomes, development of dependable, secure, and low-sodium containing potable water, together with the improvements in MAR, should be executed, and evaluated in “real-life” salinity settings.

The article is aimed identifying the impacts of DWS on the coastal areas of Bangladesh by constructing the Driver-Pressure-State-Impact-Response (DPSIR) framework based on available literature review. The article is structured first by describing the DPSIR framework theory at Section 1.1, followed by methodology employed in Section 2. Section 3 describes results and discussions, in which Section 3.1 describes the construction of DPSIR framework inputs in detail. Section 3.2 describes the health impacts of DWS and how it affects the health status of men, women, and children in particular. In addition, this article also identifies the available intervention mechanisms applied in the coastal communities and recognizes the effectiveness among the users in Section 3.3.

### 1.1. The Theory of DPSIR Framework

**Figure 1:**
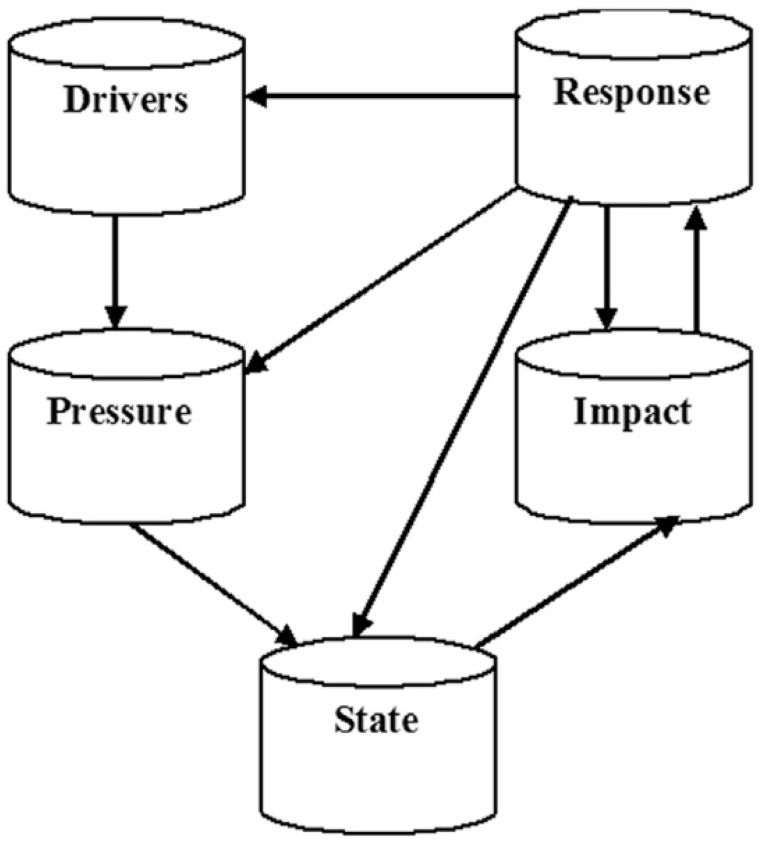
The Driver-Pressure-State-Impact-Response (DPSIR) framework [22].

While there are several theoretical contexts to demonstrate associations between the anthropogenic stresses and scenario deviations in marine and coastal ecosystems, the DPSIR framework is the most extensively applied framework [19]. The DPSIR framework was originally developed from the Pressure-State-Response (PSR) framework by Rapport and Friend in the late 80′s [20]. Later, the DPSIR framework was adapted and largely promoted by the Organization for Economic Cooperation and Development (OECD) and the European Environmental Agency (EEA) for its environmental reporting [21]. This framework is an adaptive management tool used for analyzing environmental problems by establishing a cause-effect relation between anthropogenic activities and their environmental and socio-economic consequences [22]. It is policy-oriented and provides a framework for categorizing a problem domain along the cause-effect chain [19]. Adopting a DPSIR approach can help stakeholders to identify and structure challenges in coastal systems, and use the framework to support policy and management outcomes [20]. According to the DPSIR framework there is a chain of causal links starting with ‘driving forces’ (economic sectors, human activities, etc.) through ‘pressures’ (emissions, wastes, etc.) to ‘states’ (physical, chemical, and biological). These further lead to ‘impacts’ on ecosystems, human health, and functions, eventually leading to political ‘responses’ (prioritization, target setting, indicators). Describing the causal chain from driving forces to impacts and responses is a complex task, and tends to be broken down into sub-tasks, e.g., by considering the pressure-state relationship [21] (Figure 1).

## 2. Methodology

**Figure 2:**
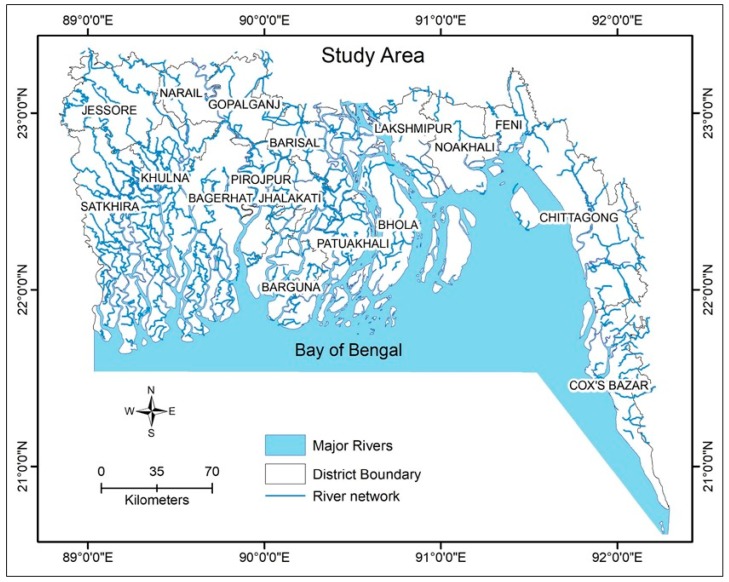
The study area: coastal regions of Bangladesh.

We constructed a DPSIR framework of DWS based on the review of literature and published case studies on the coastal regions of Bangladesh. The map of coastal regions of Bangladesh is shown in Figure 2. We further analyzed available secondary salinity data and surveyed the effectiveness of the interventions.

### 2.1. Review Protocol

**Figure 3:**
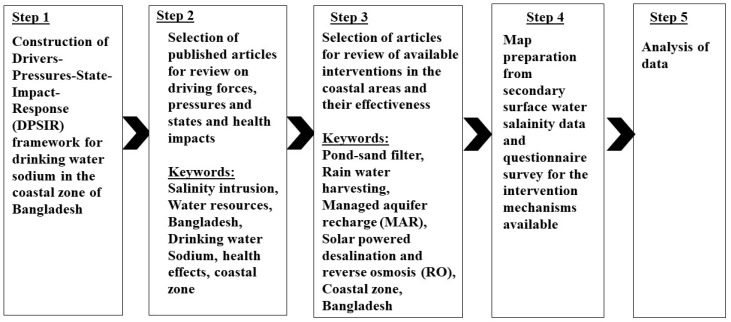
Review protocol steps and keywords of the present study.

This section is a comprehensive review of the available literature concerned with the impacts of salinity intrusion in the community health in order to construct the DPSIR framework. When we discuss the salinity intrusion in coastal areas, it automatically leads to the key point “Drinking or potable water salinity”, which is mainly caused by the presence of sodium in the drinking or potable water. The review is based on the published research through the use of scientific databases, such as Google Scholar, Web of Science, ScienceDirect, Scopus, etc. We used keyword-based screening according to the texts in the abstracts and considered articles published in English. Unpublished data such as our own previous works were used as supporting material. The review protocol steps are shown in Figure 3.

### 2.2. Inverse Distance Weighting (IDW) for Surface Water Salinity Map Preparation

To understand the area-wide distribution of salinization over two seasons in the study area, we used the IDW method for the spatial analysis of salinity data that we collected in earlier studies. We used ArcGIS version 10.1 to prepare the map. IDW uses the measured values surrounding the prediction location; wherein, greater weight is assigned to the points in proximity to the prediction location. The general formula for IDW can be presented as the following Equation (1).

(1) $$\hat{z}\left(x_{0}\right)=\overset{n}{\underset{i=1}{\sum }}\lambda_{i}z\left(x_{i}\right)$$

where $\hat{z}$ is the estimated value of an attribute at the point of interest $x_{0}$, z is the observed value at the sampled point $x_{i}$, $\lambda_{i}$ is the weight assigned to the sampled point, and n represents the number of sampled points used for the estimation. The attribute is usually called the primary variable, especially in geostatistics. To ensure that the estimates are unbiased, the sum of the weights $\lambda_{i}$ must be equal to one. The formula to determine the weights $\lambda_{i}$ can be presented in Equation (2).

(2) $$\lambda_{i}=d_{i0}^{-p}/\overset{n}{\underset{i=l}{\sum }}d_{i0}^{-p}$$

wherein, $d_{i0}$ is the distance between the sample and prediction points. As the distance becomes larger, the weight is reduced exponentially by a power parameter of p. Therefore, IDW interpolation produces a relatively rough surface, which is dependent on the distance between sample points [23]. In this research, power parameters (p) of 1 and 2 were used to provide a basis to compare the effect of different power parameters.

### 2.3. Questionnaire Survey

We obtained user opinion on rainwater harvesting and solar powered desalination plant from Nilganj and Khaprabhanga Union of Kalapara Upazila, Patuakhali District through a questionnaire survey. Patuakhali District is one of the severely salinity-affected area from the south-central part of Bangladesh. Rainwater harvesting and a solar powered desalination plant in a pilot scale is currently operating in the area. We have randomly collected 120 user opinions according to the method suggested by Cochran, taking 95% of confidence level and a 10% margin of error [24].

## 3. Results and Discussions

### 3.1. The Construction of DPSIR Framework Inputs

**Figure 4:**
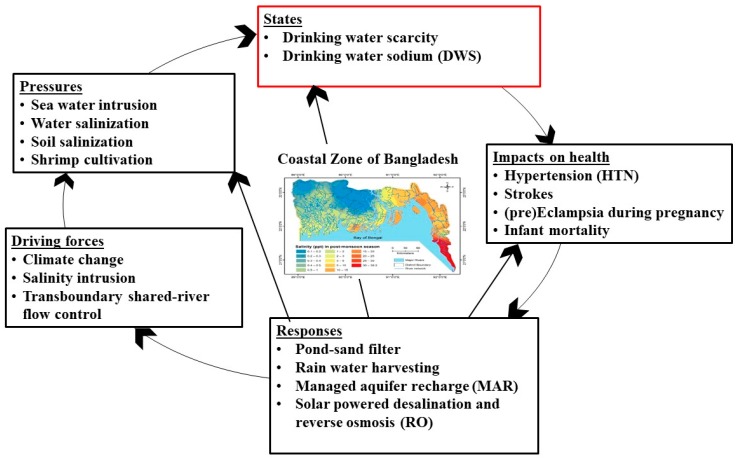
Drivers, Pressures, State, Impact, Response (DPSIR) model for drinking water sodium (DWS) in the coastal areas of Bangladesh (this study).

The application of the DPSIR framework is substantially pertinent to link the existing gaps between the scientific disciplines and the sciences of the coastal development strategy, as well as the integrated coastal zone management. However, the existing applications of the DPSIR framework in the coastal zones have been inadequate and novel innovative approaches are required to apply the model [20]. DPSIR models of coastal systems have been largely used to support and develop conceptual understanding of coastal social-ecological systems and to identify drivers and pressures in the coastal environment [20]. The framework can link the marine aquatic ecosystems to the neighboring terrestrial ecosystems through numerous flexible-structured schemes and can be applied in various geographic locations [19]. Our DPSIR framework was focused on DWS and its associated health problems to apply it in the coastal zone management policy of Bangladesh (Figure 4). The selection of input parameters to prepare the components are further discussed in the subsequent sections.

#### 3.1.1. Driving Force for Salinity Induced Health Issues in Coastal Bangladesh

**Figure 5:**
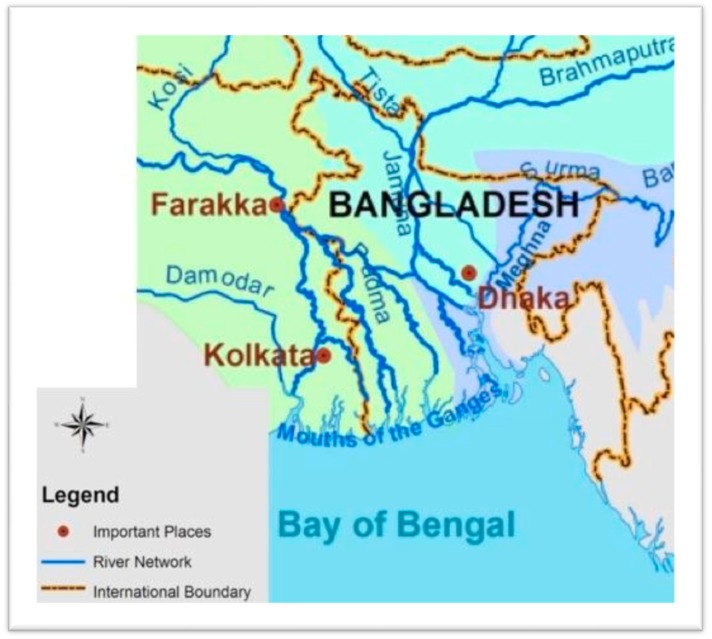
The regional transboundary rivers including the Ganges basin in Bangladesh and the Farakka Barrage built in the upstream of the Ganges (In Bangladesh the river is known as Padma). The map is modified after Baten and Titumir [26].

Drinking water sources in the coastal regions of Bangladesh vary widely. Potable water shortage is seasonal, occurring during the dry season between November and early May. Owing to less precipitation and diminished river flow, the saltiness of surface water bodies such as waterways and canals increases through dry season. Furthermore, during the dry season, ponds repeatedly dry up, leaving the coastal population with few or no options but to use hand pump-extricated saline groundwater for drinking and cooking. Whereas, amidst the monsoon season (May to October), the coastal population generally gather rainwater through household- or community-level rainwater harvesting schemes [25]. The Ganges, Brahmaputra and Meghna (GBM) basins are shared between Bangladesh and India [26]. Subsequently, the driving forces of DWS were identified to be climate change induced salinity problem and flow control of shared transboundary rivers in the upstream of Farakka Barrage in the Ganges, and others (Figure 5), which are discussed in the subsequent parts.

The intrusion of salinity into surface water and aquifers poses a significant risk to people’s access to potable water [2]. The likely effects of climate change on coastal zone districts are comprised of regular inundation by the Bay of Bengal, increased storm surges, damage of coastal swamps and wetlands; as well as expanded salinity amid the monsoon season due to the coastal tides going into rivers and estuaries [2,27]. Moreover, ascendant or lateral movement of the groundwater within the post-monsoon season, as well as the direct flooding of the saline or brackish water for shrimp cultivation, are anthropogenic causes of salinization in the south [2].

Investigation of spatial and temporal inclination of surface water salinity in the coastal zones of Bangladesh showed that the maximum salt encroached areas during the monsoon season are: (i) Khulna, Satkhira, Bagerhat, Jessore, and Gopalganj—districts located in the extreme southwestern zones; (ii) Bhola, Noakhali, and Feni—districts positioned in the lower Meghna River floodplain and the Meghna estuarine floodplain; (iii) Chittagong and Cox’s Bazar—districts located in the southeastern portion of the Chittagong coastal plains adjacent to the Bay of Bengal; and (iv) Barisal, Jhalkathi, Patuakhali, and Barguna—the slightly saline diverged mid-south zone districts. All these districts are affected during the dry season [28]. Increased anions and cations such as Cl^−^, Na^+^, SO_4_^2−^, HCO_3_^−^, etc. throughout the post-monsoon season shows the inward movement of saltwater [28]. The extreme seasonal variation of anions and cations is, therefore, ascribed to the upstream freshwater withdrawal in the Farakka barrage, India, which plummets water currents in the downstream followed by inland intrusion of seawater [28,29]. The more prominent the entrance of marine impact into the terrestrial inland, the lower the accessibility of freshwater, wherein its combination with salt water results to brackish quality [30]. Along with their contribution to the total dissolved solids (TDS) and the electrical conductivity (EC), the abundance of the key anions and cations in the surface water was confirmed in the subsequent order of Na^+^ > Ca^2+^ > K^+^ > Mg^2+^ and Cl^−^ > SO_4_^2−^ > HCO_3_^−^ > NO_3_^−^ > CO_3_^2−^ [28].

**Figure 6:**
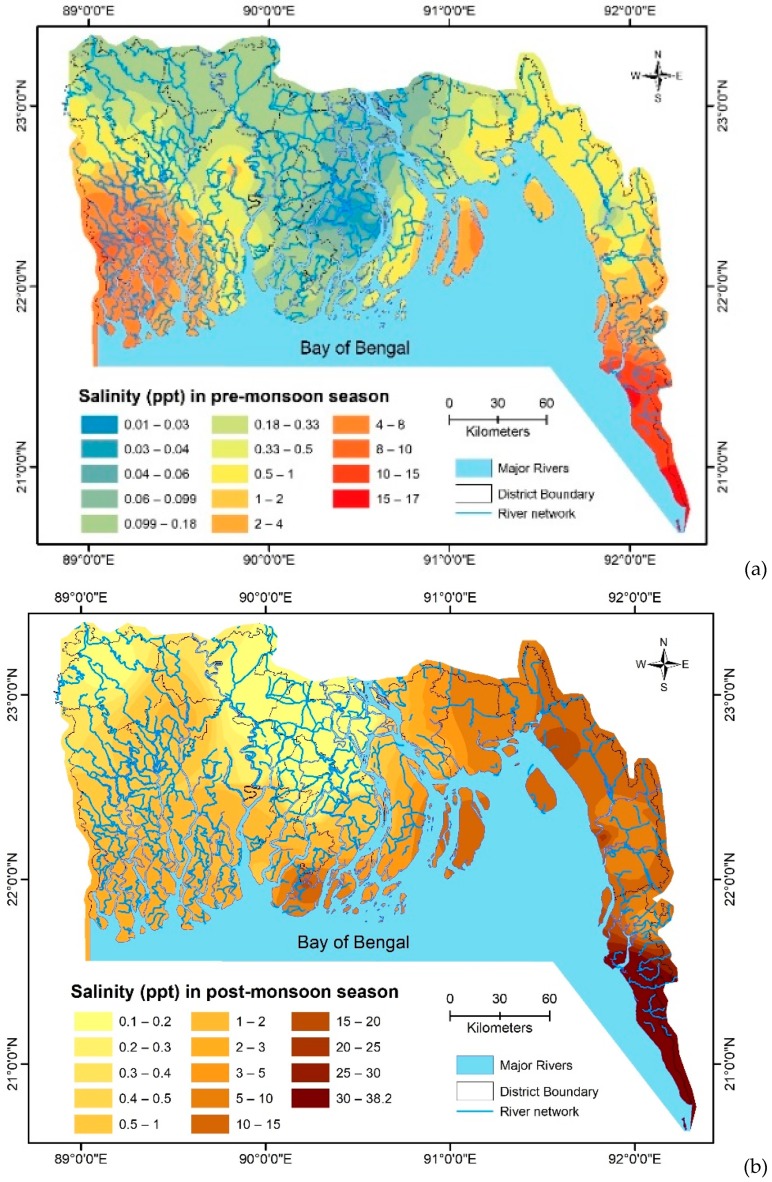
Spatial salinity distribution in parts per thousand (ppt) in the pre-monsoon season (**a**) and post-monsoon season (**b**) in Bangladesh’s coastal rivers and estuaries. Pre-monsoon (n = 96) and post-monsoon (n = 44) sampling points (this study).

The spatial and temporal variation of surface water salinity data collected from the secondary sources were incorporated and the following map was generated (Figure 6). The critical seasonal difference in the salinity of the pre-monsoon (Figure 6a) season, and the salinity outcome of the post-monsoon season (Figure 6b), in the surface water of Bangladesh is easily distinguishable.

As described in the earlier section, Gopalganj Sadar Upazila is one of the coastal districts of Bangladesh that is most affected by salinity. The risk of salinity was investigated for the surface water (ponds and river) and the groundwater samples, from shallow tube wells (STW) and deep tube wells (DTW). The water samples were collected from various irrigation sources in the pre-monsoon season in the month of March 2012. The depth of the STW varied from 100 to 150 feet, while the depth of DTW was more than 600 feet [31]. Correlation matrices and principal component analysis (PCA) on both surface and ground water established higher EC and TDS in relation to salinity stress due to Na^+^, K^+^, Cl^−^, and higher total hardness (TH) in relation to Ca^2+^, Mg^2+^, PO_4_^3−^, and SO_4_^2−^ [30]. Islam et al. further examined 46 groundwater samples of Gopalganj district (23 post-monsoon and 23 pre-monsoon periods) ranging from the depth of 48 m to 200 m [32]. Hydrochemical analysis revealed groundwater in this area was neutral to slightly alkaline and dominating cations were Na^+^, Mg^2+^, and Ca^2+^, along with major anions Cl^−^ and HCO_3_^−^. The spatial distribution of irrigation water quality index (IWQI), electrical conductivity (EC), soluble sodium percentage (SSP), and total hardness (TH) during both seasons confirmed the influence of salinity from the sea. The existing low-flow in the major river system was the driving factor of overall groundwater quality in the study area [31].

Rahman et al. further evaluated the likely human risk of consuming the said groundwater samples to adults and children. Maximum hydrochemical parameters corresponding to pH, EC, TDS, Na^+^, Cl^−^, HCO_3_^−^, As, Mn, Fe, B, NO_3_^−^, CO_3_^−^, etc. surpassed the limits of various potable water standards. The spatial distribution of EC, Cl^−^, As, Fe, and Mn varied considerably in both pre-monsoon and post-monsoon seasons. From multivariate statistical analysis, high-salinity characteristic of groundwater resulting from seawater intrusion was observed. Strong, moderate, and weak spatial dependences are found for both seasons. The exponential semivariogram model was found to be overriding when considering the best-fit model for both seasons. The exponential semivariogram model is a geostatistical function often used to recognize the spatial distribution of specific water-quality parameters for a specified geographic area. Furthermore, mean values of the hazard quotient (HQ, i.e., carcinogenic or non-carcinogenic health risk based on quantification of risk level for metal or metalloid exposure) and hazard index (HI, i.e., which is the sum of all calculated HQs used, to assess the overall potential for non-carcinogenic effects possessed by the calculated chemicals) was constructed for As, Fe, Mn, B, NO_3_^−^, and F^−^. The HQ and HI values of the aforementioned chemicals in the groundwater samples varied seasonally and carries a considerable health and well-being hazard to adults and children [33].

Molar proportion of the Cl^−^/Σ(anions) and Na⁺/Na^+^+Cl^−^ specified that the groundwater in the south-central part of Bangladesh, such as the Barguna and Patuakhali districts, was affected by the intrusion of seawater. The groundwater of Barguna and Patuakhali was clearly dominated by the cations and anions of Na^+^, Mg^2+^, Ca^2+^, Cl^−^, and HCO_3_^−^ in both wet and dry monsoon seasons [34]. While the maximum sodium intake according to WHO guideline 2011 [35] is 200 mg/L, the Na^+^ concentrations in the groundwater were reported to be 863 mg/L and 825 mg/L in the pre-monsoon and post-monsoon seasons, respectively [36]. In another study, 18 groundwater samples and 2 pond water samples were collected from the tube wells of Chittagong district, located in the Southeastern coastal region of Bangladesh. The depths of the tube wells varied from 244 to 365 m and the tests indicated that the 75% of the groundwater samples were comprised of Na^+^. In addition, the average salinity of the shallow tube wells ranged from 5.11 to 6.48 dS/m, while the pond water salinity ranged from 0.11 to 3.12 dS/m [37]. It is, therefore, clear from the above recent studies that the salinity of the surface water, as well as the groundwater resources, in the coastal districts varied significantly. The level of salinity within the shallow and deep tube wells varies with respect to the depth of the wells and the distance from the Bay of Bengal. Along with this, the groundwater in the coastal districts was categorized with a higher level of non-carcinogenicity and higher level of vulnerability to arsenic and other elements leading to carcinogenicity.

#### 3.1.2. Pressures and State Induced Health Issues in Coastal Bangladesh

Pressures identified in the framework were seawater intrusion, salinization of soil and water resources, as well as shrimp cultivation in the coastal zones; thereby leading to the state of drinking water scarcity and presence of sodium in drinking water. Because of the microbial contamination of surface water and uneven distribution of the water resources, the rural population depends heavily on tube wells for drinking water [14]. In these rural areas, more than 1.2 million hand-pumped tube wells were installed by the Government of Bangladesh (GoB) and six times as many have been installed by private individuals, nongovernmental organization (NGOs), and other agencies. There are certain areas in the coastal districts where both shallow and deep tube wells are not useful due to high salinity in groundwater [29]. It is, therefore, clear that saltwater invasion and salinization are contributors to consumable water shortages on coastal areas (both regionally and globally), which forces people to depend on alternative sources for water consumption [38]. The sources of consumable water include deep and shallow groundwater from aquifers, small ponds with or without the pond sand filters (PSF, sand, and gravel filters), collected rainwater, bottled water, streams, etc. [25,39]. Groundwater in coastal Bangladesh is no longer a safe source for drinking water collection.

#### 3.1.3. Impacts and Responses Induced Health Issues in Coastal Bangladesh

Coastal communities in Bangladesh had little consciousness of the dangers related to the excessive salt consumption from their food. Moreover, reducing salt intake tactics were not very important to them [5]. Impacts of excessive salt on health includes hypertension or high blood pressure in both male and female adults, leading to higher risks of stroke. Pregnant women have been found to be particularly at risk of gestational hypertension, (pre)eclampsia, and post-partum infant morbidity and mortality. Major interventions to reduce drinking water sodium in practice are identified as pond-sand filter (PSF), rainwater harvesting, and managed aquifer recharge (MAR). Advanced and expensive potable water solutions such as solar-powered desalination plants, reverse osmosis, and electrodialysis machines are amongst the options to reduce salinity levels in water. These methods were also reported in some pilot scale studies aimed to yield safe consumable water. Along with this, government organizations and NGOs have also reinforced public endeavors to manage salinity issues by emphasizing mutually communal adaptation strategies and institutional efforts [2]. This article further outlines DWS and the combined community-based methods intended to ease the potable water shortage in the coastal region of Bangladesh. Assuming that the coastal people in Bangladesh avoid consumption of highly saline drinking water, it is still projected that the exposure to salinity will further rise as an outcome of climate change, sea level rise, and various other environmental stimuli [40]. Thus, it is crucial to assess and promote reasonable approaches to provide affordable and accessible potable water with little or no salt content.

### 3.2. Health Impacts: Insights of Coastal People

Potable water security is one of the principal concerns for the well-being and sustainable development of the residents living within the coastal zones [2]. While the direct and indirect intrusion of salinity in fresh groundwater affects human well-being [37], its serious implications on population health must be clearly understood [41]. Salt was depicted as a fundamental component of nourishment with strong cultural and religious roots. People described both health benefits and risks of salt ingestion. The sources of dietary salt, people’s belief and perceptions, as well as practices associated with salt consumption, are particularly important among the coastal population of Bangladesh who are at high risk of hypertension owing to exposure to climate change-induced environmental salinity [5]. In 2011, a comprehensive risk analysis for disproportionate salt ingestion was conducted among the respondents of the Chakaria sub-district, a rural area on the southeastern coastal region of Bangladesh. A cross-sectional mixed method study was done by focus group discussions (FGD), key informant interviews, and 400 adult interviews. The respondents believed that the cooking procedure made the salt harmless. The overall risk perception regarding excessive salt consumption was low among the respondents and they were not aware that salt can naturally occur in both food and water [5]. Jabed et al. identified the public discernment on the water salinity effects on human health in the Chittagong South-Eastern coastal region. Owing to the use of saltwater, villagers suffered from numerous diseases including skin ailments, hair fall, diarrhoea, gastric diseases, and high blood pressure (Table 1) [37]. The neglected existence of salt in potable water leads to a risky level of salt ingestion among the population. Such excessive salt intake, in turn, can put a high number of individuals including pregnant women at a risk of hypertension and even death [5].

#### 3.2.1. Impacts on Maternal Health

Drinking water sodium has serious implications for the health of the community, particularly for pregnant women. People exposed to slightly saline (1000–2000 mg/L) and moderately saline (≥2000 mg/L) concentrations of drinking water had, respectively, 17% (*p* < 0.1) and 42% (*p* < 0.05) higher chance of being hypertensive than those who consumed freshwater (<1000 mg/L). It was further found that females have a 31% higher chance to be hypertensive than the males. In addition, interviewees 35 years old and above have approximately 2.4 times higher risk of hypertension compared to the interviewees under 35 years. Moreover, it was found that for ages 35 years and above, pre-hypertension and hypertension risks were 53.8% higher for slightly saline water and 62.5% higher for moderate saline water. In total, the disease risks in saline water-exposed groups were 50.1% higher than national rural statistics. For moderate salinity exposure, the pervasiveness of hypertension among the interviewees was 21%, 60%, and 48% higher than national statistics (23.6%), respectively. Although there was a slight periodical difference in the salinity of potable water, the dry season exhibited an increasing trend of salinity and maximum levels of blood pressure. Average salinity, as well as the relevant pervasiveness of hypertension, were greater among the consumers of deep aquifer water (21.6%) than for the users of shallow aquifer waters (20.8%) [15].

**Figure 7:**
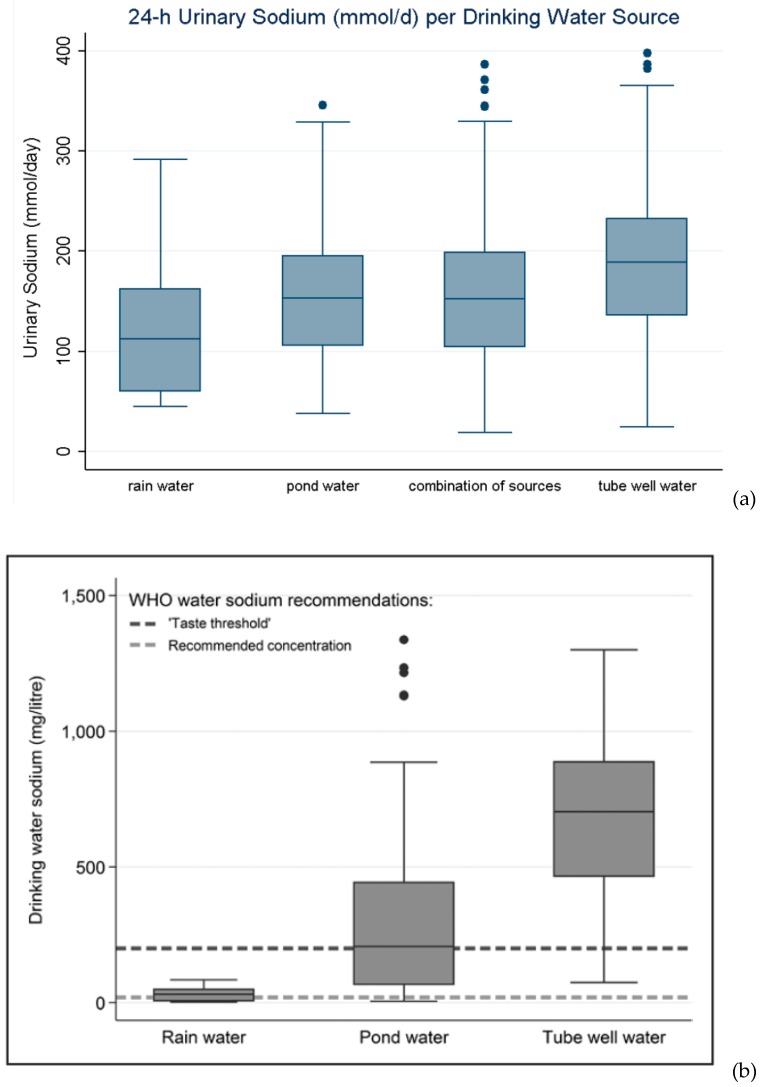
(**a**) Urinary sodium excretion (mmol/day) of healthy pregnant women sampled in the dry season (n = 645) [4] and (**b**) Dry season DWS concentrations measured in each by water source type in Dacope 2009 to 2010 (mg/L) [42].

To understand the effect of exposure to DWS on maternal health in the coastal areas, information was obtained from different types of studies. The most notable types of information were water sources and water consumption types (shallow tube well, groundwater, rainwater consumption), daily urinary sodium concentration of the pregnant women, seasonal variations (dry or wet), and hospital incidence due to eclampsia or (pre)eclampsia, etc. Note that (pre)eclampsia and gestational hypertension is a condition that occurs only during pregnancy and is characterized by one or more convulsions and high blood pressure; while eclampsia is the severe complicated form of (pre)eclampsia. Khan et al. identified an estimated average DWS of 5–16 g/day on dry season compared to 0.6–1.2 g/day on wet season. The daily sodium concentration in the urine was found to be 3.4 g/day (range 0.4 to 7.7 g/day) [4]. The women who relied on shallow tube wells for potable water were more likely to have urine sodium concentration of >100 mmol/day compared to the women consuming rainwater (odds ratio (OR) = 2.05; 95% confidence interval (CI), 1.11–3.80) (Figure 7a). The yearly hospital incidence of hypertension amid the pregnancy period was greater within the dry season (OR = 12.2%; 95% CI, 9.5–14.8) than within the wet season (OR = 5.1%; 95% CI, 2.91–7.26) [4]. A substantial relation between drinking water sodium and (pre)eclampsia and gestational hypertension was distinctly delineated (Table 1) [40]. It was clearly found in the Dacope Upazila of Khulna District, that the potable water sources had remarkably high-levels of sodium concentration (mean 516.6 mg/L, ±524.2) (Figure 7b). The women dependent on the tube-well or groundwater were more susceptible to disease risk than the rainwater consumers (*p* = 0.001). Adjusted risks for (pre)eclampsia and gestational hypertension considered together increased in a dose-response manner for increasing sodium concentrations (300.01–600 mg/L, 600.1–900 mg/L, 900.01 mg/L, compared to 300 mg/L) in drinking water (ORs 3.30 (95% CI 2.00–5.51), 4.40 (2.70–7.25), and 5.48 (3.30–9.11), (*p* trends to 0.001)) [40].

#### 3.2.2. Drinking Water Sodium and Hypertension

Consequent data gathering and review on the pressing topic found that DWS concentrations were significantly associated with high blood pressure (hypertension) (Table 1). It was found that systolic and diastolic blood pressures were typically lesser by 0.95/0.57 mmHg for each 100 mg/L decline in DWS and the probability of hypertension was decreased by 14% [15]. Scheelbeek et al. examined the effects of DWS on pregnant women’s blood pressure (gestational hypertension). The author showed that increased blood pressure can have a serious impact on maternal and fetal health. Comprehensive linear mixed regression models further examined the relationship of systolic and diastolic blood pressure and drinking water sources of healthy women. Subsequent tuning of several other factors revealed that consumers of water from high saline sources (tube-wells and pond water) had expressively higher average systolic (+4.85 and +3.62 mmHg) and diastolic (+2.30 and +1.72 mmHg) blood pressure than the rainwater consumers [38]. Analogous pattern of drinking water sources were also observed on 24-h urinary sodium (mmol/day) data. Higher concentration of DWS from salt water contamination was linked with higher blood pressure in normotensive (or normal blood pressure) pregnant women in the coastal regions. DWS levels may also vary throughout the pregnancy stage. Differences may also be induced during pregnancy when a woman is required to switch their source of potable water from rainwater-based system to a higher-saline substitute source due to the depletion of water storage [42].

#### 3.2.3. Drinking Water Sodium and Newborn Mortality

DWS could be a major factor on infant and newborn death in coastal Bangladesh (Table 1). Although not much data was available in this area, Dasgupta et al. (2016) used a probit and logit model on Bangladesh Demographic and Health Surveys (BDHS) data from 2004 and 2007 to evaluate the likelihood of mortality for newborns under two months of age. Subsequently, the BDHS data were spatially extrapolated on the monthly soil salinity data from 2001–2009. Since the household-specific drinking water salinity was not measured, soil data that fell within the 40 km of the BDHS clusters were considered [27]. The study examined multiple factors of infant mortality and taking the last stages of gestation into consideration, it was found that exposure to saltwater was highly significant to infant mortality. On the other hand, it was found that saltwater ingestion in the earlier months of gestation was not significant. The implications of DWS on infant mortality were found to be dependent on a variety of factors such as the maternal stage of development, education, gender of the household head, assets, toilet facilities, potable water sources, cooking fuels, etc. The model by Dasgupta et al. strongly suggested that DWS is a major factor to infant and newborn mortality in coastal Bangladesh and gave a new insight into the relationship between post-natal influences and the pacing of prenatal ingestion of DWS [27].

### 3.3. Interventions to Decrease Drinking Water Sodium (DWS): Are These Interventions Effective?

Responses obtained from the DPSIR framework were taken as interventions. Several interventions have been implemented to tackle the shortage of potable water owing to salt intrusion in the coastal regions of Bangladesh. With varying success, southwestern Bangladesh communities currently use drinking water via the following interventions: rainwater harvesting, ponds, pond sand filters, managed aquifer recharge (MAR), tube wells, etc. [43]. From the questionnaire survey we obtained user opinions regarding established rainwater harvesting practices and a very new solar-powered desalination system reverse osmosis (RO), which is still at the pilot scale.

#### 3.3.1. Rainwater Harvesting

Rainwater harvesting is an ancient technique for collecting water especially when the water scarcity is intense. This technique has been modified by different NGOs that provide drinking water to the needy people of coastal areas of Bangladesh at an affordable cost. For instance, SPS Khulna, an NGO, set up concrete tanks that are used for rainwater harvesting, which costs 15,000 BDT ($180 US) per family, while some locally made large earthen containers (local name motka) costs only 1000 BDT ($12 US). In the questionnaire survey, it was found that 50% of the respondents in the Kalapara Upazila of Patuakhali District Bangladesh reported rainwater harvesting to be a moderately effective measure, while 20% and 23% respondents thought it was a very effective and highly effective measure, respectively. Consuming rainwater lessens the risk of salinity exposure through lowered sodium intake and is beneficial to cardiovascular health of the coastal communities. However, it also decreases the ingestion of vital cardio-protective minerals such as magnesium and calcium [25]. WHO recommends the addition of essential elements such as calcium and magnesium to mineralize the desalinated water in order to safeguard consumers from cardiovascular risks [44]. Therefore, improvement of the rainwater is suggested, through remineralization of the important elements [25].

#### 3.3.2. Desalination and Reverse Osmosis (RO)

Desalination systems remove or reduce salts from saline water (either seawater or brackish groundwater) [45]. Examples of these are pressure-driven membrane processes such as reverse osmosis (RO) and nano-filtration. These processes are primarily driven by mechanical energy, usually in the form of electrically powered pumps [45]. RO is now gradually being more used to provide fresh drinking water under the condition of freshwater scarcity in many parts of Bangladesh. It is a new source of freshwater that will not diminish during the times of drought, thus securing safe water supply all year round. RO technology is used to eradicate dissolved solids, color, organic contaminants, and pollution from raw water. The RO system is a more technical and economically feasible drinking water source compared to the other technologies of the study area. The system can also play a great role in a disaster risk reduction (DRR) based program solution of drinking water shortages by building water security related resilience in coastal household level in Bangladesh [46]. Although the potential of desalination plants such as RO require very high cost and energy, it is still in the pilot scale level in some parts of coastal Bangladesh. During the questionnaire survey it was found that a local NGO installed four solar-powered desalination RO plants in Kalapara Upazila, of Patuakhali District Bangladesh, which is one of the severely salinity affected coastal districts mentioned earlier. Here, most of the respondents (65.8%) thought that RO was not effective at all, whereas 12.5% think it was not effective and 6.7% think it was a moderately effective measure.

#### 3.3.3. Managed Aquifer Recharge (MAR)

**Figure 8:**
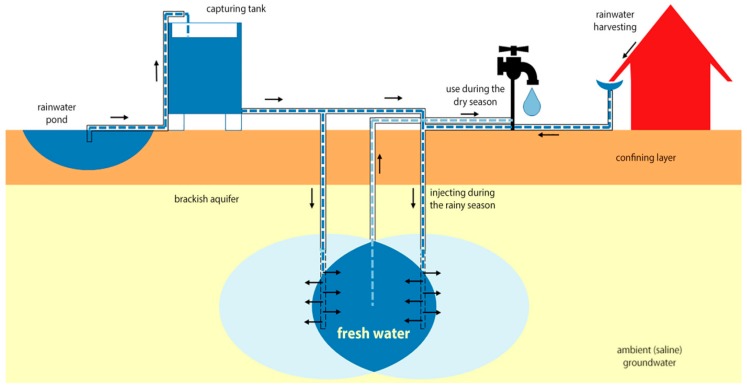
Schematic overview of a managed aquifer recharge (MAR) system in South-west Coastal Bangladesh [15].

MAR can be applied as a measure to reverse and prevent coastal aquifer salinization related to the overexploitation of groundwater and water table decline [47]. The quality of the produced water, available for drinking or irrigation processes, is strongly dependent on the aquifer’s hydrogeochemical characteristics and on the MAR system design and operation [48]. MAR is the purposeful recharge of water to aquifers for subsequent recovery or environmental benefit. It involves methods such as riverbank filtration, stream bed weirs, infiltration ponds, and injection wells in order to increase groundwater storage [47]. Integrated holistic approaches such as the rainwater harvesting systems and PSF near rainwater-supplied surface ponds, and rainwater-supplied MAR, are being upgraded from a single rainwater or PSF system. These structures capture available rainwater during the monsoon and stock it for imminent usage in the dry season (Figure 8). While these interventions may be effective in reducing DWS exposure [25], MAR is a promising adaptive approach for increasing the accessibility of salt free potable water that sustains almost a yearly water supply. Since MAR storage of rainwater occur under confined conditions, it is further secured from evaporation as well as strong to tidal storms, cyclones, and saltwater permeation (Figure 8) [38,49]. In a MAR system, a freshwater lens is purposefully created amidst the brackish aquifers, to supply surface freshwater or rainwater to the aquifers and bring the hydrological equilibrium. It is a likely to be a key solution for the salinity problem in the southwest coastal Bangladesh [38].

However, a recent study undertaken in the southwestern coastal region of Bangladesh by Scheelbeek et al. (2017) reported that a gradual increase in sodium concentration over the course of the dry season was observed in MAR. Median sodium concentrations of the pond and MAR sources were around 400 mg/L toward the end of the dry season, whereas median sodium concentrations in tube wells exceeded 800 mg/L, followed by extremes of >1500 mg/L [15]. Some rainwater users mixed their rainwater with water from other sources to prolong the period of rainwater use. Toward the end of the dry season, only those with large amounts of storage space (and hence more likely to consume unmixed rainwater) still reported rainwater as the main drinking-water source, which explains the high outliers in sodium concentrations in “rainwater” in the early dry-season measurements [15]. MAR can be affected by many risks. Those risks are related to different technical and non-technical aspects of recharge, like water availability, water quality, legislation, social issues, etc. [50]. It is, therefore, very important to build community participation and awareness regarding the maintenance and successful operation of MAR. MAR users should be trained and be aware that they should not mix saline water or water from other sources to prolong the uses of MAR in the dry season.

## 4. Conclusions

DWS is a pressing issue in the present socio-economic, environmental, and climatic condition in the coastal region of Bangladesh, where salinity and seawater intrusion are serious issues. Here, we reviewed the salinity scenario of Bangladesh and constructed the DPSIR framework of DWS. We further reviewed the health impacts of high salinity-contaminated potable water on local people’s health, maternal health, hypertension, and infant mortality in the coastal population of Bangladesh. Several key intervention mechanisms were also recognized to manage the saline water. The list of issues is inevitably extensive such as infant mortality, hypertension among adults, and (pre)eclampsia among pregnant women, which leads to millions of people being affected by heart diseases. Several interventions, such as rainwater harvesting, PSF system, MAR usage, and the integration of mixed sources, were reviewed on the content of DWS. While rainwater has the positive impact of low or no sodium intake on human health, it still possesses harmful impacts on human health for not containing essential micronutrients such as calcium and magnesium. Meanwhile, a gradual increase in sodium concentration over the course of the dry season was observed in MAR and is a serious risk factor for MAR failure. Similarly, RO and other desalination technologies are still in the pilot scale study, which requires further exploration to apply to the mass scale. It is very important to identify the mechanisms by which the above-mentioned interventions may fail. Moreover, it is crucial for coastal people to have increased consciousness regarding DWS and to be involved in the maintenance of the technologies which could aid in the desalinization of water.

## Figures and Tables

**Table 1:** DWS and its effects and associated health impacts reported from different coastal regions of Bangladesh.

Effects and Health Impacts Reported	Data Collection	Types of Sampling	Location	Ref.
Skin diseases, hair fall, diarrhoea, gastric and high blood pressure (BP)	Household random sampling (2016–2017)	Peoples’ perception, 153 households	Two selected villages of Chittagong city corporation	[37]
Drinking water salinity and blood pressure measurements	DWS sampling, information on food intake and BP	1500 households	21 unions from 9 coastal districts	[41]
People’s perception, practice and belief in the intake of salt and health risks in Bangladesh, vulnerable to climate change	Cross-sectional mixed method study between April–June 2011	6 focus group discussions (FGD), 8 key informant interviews (KII), 60 free listing exercises, 20 ranking exercises, 10 observations, and 400 questionnaire survey of adults	Chakaria, Southeastern coastal region of Bangladesh	[5]
The effect of DWS on pregnant women’s BP	Data on BP, potable water source, personal lifestyles, and environmental factors between January 2009 to June 2010	701 expectant females	Dacope, Khulna district, Southwestern coastal region	[42]
The effect of DWS on the BP	DWS, BP, and information on personal lifestyles, and environmental factors	581 expectant females	Dacope, Batiaghata and Paikghaccha; Khulna. Southwestern coastal region	[15]
The relationship of MAR water on BP	Participants’ source of drinking and cooking water; salinity level and EC of household stored water; BP and urinary sodium and protein measurements	A stepped-wedge cluster-randomised controlled community trial design; 16 communities over five monthly visits	Coastal regions of Bangladesh	[38]
DWS to elucidate the periodical pattern of hypertension	Water salinity data (1998–2000); Drinking water sources, 24-h urine samples, BP (October 2009–March 2010). The hospital data on the occurrence of hypertension amid gestation among 969 expectant females (July 2008 through March 2010)	343 expectant females	Dacope Upazila, Khulna. Southwestern coastal region	[4]
DWS and the risk of (pre)eclampsia and hypertension during pregnancy	Case control study; epidemiological and clinical data; urinary sodium and sodium concentrations in drinking water	202 expectant females with (pre)eclampsia or gestational hypertension	Dacope Upazila, Khulna. Southwestern coastal region	[40]
The post-natal impact of pre-natal salinity exposure	Bangladesh Demographic and Health Surveys (BDHS) for 2004 and 2007, monthly soil salinity data for 2001–2009; spatial interpolation of infant mortality that lie within 40 km of the BDHS clusters	DWS consumed during gestation lead to hypertension, (pre)eclampsia and post-partum infant mortality	Four coastal regions of southern Bangladesh: Barisal, Chittagong, Dhaka and Khulna	[27]

## References

[1] Werner A.D., Bakker M., Post V.E.A., Vandenbohede A., Lu C., Ataie-Ashtiani B., Simmons C.T., Barry D.A. (2013). Seawater intrusion processes, investigation and management: Recent advances and future challenges. Adv. Water Resour..

[2] Abedin M., Habiba U., Shaw R. (2014). Community perception and adaptation to safe drinking water scarcity: Salinity, arsenic, and drought risks in coastal Bangladesh. Int. J. Disaster Risk Sci..

[3] Hoque M.A., Scheelbeek P.F., Vineis P., Khan A.E., Ahmed K.M., Butler A.P. (2016). Drinking water vulnerability to climate change and alternatives for adaptation in coastal South and South East Asia. Clim. Chang..

[4] Khan A., Ireson A., Kovats S., Mojumder S., Khusru A., Rahman A., Vineis P. (2011). Drinking water salinity and maternal health in coastal Bangladesh: Implications of climate change. Environ. Health Pers..

[5] Rasheed S., Siddique A., Sharmin T., Hasan A., Hanifi S., Iqbal M., Bhuiya A. (2016). Salt intake and health risk in climate change vulnerable coastal Bangladesh: What role do beliefs and practices play?. PLoS ONE.

[6] Brammer H. (2014). Bangladesh’s dynamic coastal regions and sea-level rise. Clim. Risk Manag..

[7] Rahman S., Rahman M. (2015). Climate extremes and challenges to infrastructure development in coastal cities in Bangladesh. Weather Clim Extrem..

[8] MoWR/GOB Coastal Zone Policy 2005. http://lib.pmo.gov.bd/legalms/pdf/Costal-Zone-Policy-2005.pdf.

[9] Bhuiyan M.J.A.N., Dutta D. (2012). Assessing impacts of sea level rise on river salinity in the Gorai river network, Bangladesh. Estuar Coast. Shelf Sci..

[10] Shammi M., Karmakar B., Rahman M., Islam M., Rahman R., Uddin M. (2016). Assessment of salinity hazard of irrigation water quality in monsoon season of Batiaghata Upazila, Khulna District, Bangladesh and adaptation strategies. Pollution.

[11] Halim M.A., Majumder R.K., Nessa S.A., Hiroshiro Y., Sasaki K., Saha B.B., Saepuloh A., Jinno K. (2010). Evaluation of processes controlling the geochemical constituents in deep groundwater in Bangladesh: Spatial variability on arsenic and boron enrichment. J. Hazard. Mater..

[12] Bahar M.M., Reza M.S. (2010). Hydrochemical characteristics and quality assessment of shallow groundwater in a coastal area of Southwest Bangladesh. Environ. Earth Sci..

[13] Shameem M.I.M., Momtaz S., Rauscher R. (2014). Vulnerability of rural livelihoods to multiple stressors: A case study from the southwest coastal region of Bangladesh. Ocean. Coast. Manag..

[14] Rahman M., Rasheduzzaman M., Habib M., Ahmed A., Tareq S., Muniruzzaman S. (2017). Assessment of fresh water security in coastal Bangladesh: An insight from salinity, community perception and adaptation. Ocean. Coast. Manag..

[15] Scheelbeek P.F., Chowdhury M.A., Haines A., Alam D.S., Hoque M.A., Butler A.P., Khan A.E., Mojumder S.K., Blangiardo M.A., Elliott P. (2017). Drinking water salinity and raised blood pressure: evidence from a cohort study in coastal Bangladesh. Environ. Health Pers..

[16] Islam A., Majumder A. (2012). Hypertension in Bangladesh: A review. Indian Heart J..

[17] Müller D.N., Wilck N., Haase S., Kleinewietfeld M., Linker R.A. (2019). Sodium in the microenvironment regulates immune responses and tissue homeostasis. Nat. Rev. Immunol..

[18] Nishida C., Uauy R., Kumanyika S., Shetty P. (2002). The Joint WHO/FAO Expert Consultation on diet, nutrition and the prevention of chronic diseases: Process, product and policy implications. Public Health Nutr..

[19] Patrício J., Elliott M., Mazik K., Papadopoulou K.N., Smith C.J. (2016). DPSIR—Two decades of trying to develop a unifying framework for marine environmental management?. Front. Mar. Sci..

[20] Lewison R.L., Rudd M.A., Al-Hayek W., Baldwin C., Beger M., Lieske S.N., Jones C., Satumanatpan S., Junchompoo C., Hines E. (2016). How the DPSIR framework can be used for structuring problems and facilitating empirical research in coastal systems. Environ. Sci. Pol..

[21] Kristensen P. The DPSIR Framework. Presented at the Workshop on a Comprehensive/Detailed Assessment of the Vulnerability of Water Resources to Environmental Change in Africa using River Basin Approach, UNEP Headquarters.

[22] Gari S.R., Newton A., Icely J.D. (2015). A review of the application and evolution of the DPSIR framework with an emphasis on coastal social-ecological systems. Ocean. Coast. Manag..

[23] Burrough P.A., McDonnell R.A. (1998). Principles of Geographical Information Systems.

[24] Cochran W.G. (1963). Sampling Techniques.

[25] Naser A., Martorell R., Narayan K.V., Clasen T.F. (2017). First do no harm: The need to explore potential adverse health implications of drinking rainwater. Environ. Sci Tech..

[26] Baten M.A., Titumir R.A.M. (2016). Environmental challenges of trans-boundary water resources management: The case of Bangladesh. Sustain. Water Resour. Manag..

[27] Dasgupta S., Huq M.M., Wheeler D. (2016). Drinking water salinity and infant mortality in coastal Bangladesh. Water Econ. Pol..

[28] Shammi M., Rahman M.M., Islam M.A., Bodrud-Doza M., Zahid A., Akter Y., Quaiyum S., Kurasaki M. (2017). Spatio-temporal assessment and trend analysis of surface water salinity in the coastal region of Bangladesh. Environ. Sci. Pollut. Res..

[29] Abedin M.A., Shaw R. (2018). Constraints and coping measures of coastal community toward safe drinking water scarcity in Southwestern Bangladesh. Science and Technology in Disaster Risk Reduction in Asia.

[30] Loitzenbauer E., Mendes C.A.B. (2012). Salinity dynamics as a tool for water resources management in coastal zones: An application in the Tramandaí River basin, southern Brazil. Ocean. Coast. Manag..

[31] Shammi M., Rahman R., Rahman M.M., Moniruzzaman M., Bodrud-Doza M., Karmakar B., Uddin M.K. (2016). Assessment of salinity hazard in existing water resources for irrigation and potentiality of conjunctive uses: A case report from Gopalganj District, Bangladesh. Sust. Water Res. Manag..

[32] Islam M.A., Rahman M.M., Bodrud-Doza M., Muhib M.I., Shammi M., Zahid A., Akter Y., Kurasaki M. (2017). A study of groundwater irrigation water quality in south-central Bangladesh: A geo-statistical model approach using GIS and multivariate statistics. Acta Geochim..

[33] Rahman M.M., Islam M.A., Bodrud-Doza M., Muhib M.I., Zahid A., Shammi M., Tareq S.M., Kurasaki M. (2018). Spatio-Temporal Assessment of groundwater quality and human health risk: A case study in Gopalganj, Bangladesh. Expo. Health.

[34] Islam M., Zahid A., Rahman M.M., Rahman M.S., Islam M.J., Akter Y., Shammi M., Bodrud-Doza M., Roy B. (2017). Investigation of groundwater quality and its suitability for drinking and agricultural use in the south central part of the coastal region in Bangladesh. Expo. Health.

[35] WHO (2011). Guidelines for Drinking-Water Quality.

[36] Islam S.D.U., Majumder R.K., Uddin M.J., Khalil M.I., Alam M.F. (2017). Hydrochemical characteristics and quality assessment of groundwater in Patuakhali District, southern coastal region of Bangladesh. Expo. Health.

[37] Jabed M., Paul A., Nath T. (2018). Peoples’ perception of the water salinity impacts on human health: A case study in south-eastern coastal region of Bangladesh. Expo. Health.

[38] Naser A.M., Unicomb L., Doza S., Ahmed K.M., Rahman M., Uddin M.N., Quraishi S.B., Selim S., Shamsudduha M., Burgess W. (2017). Stepped-wedge cluster-randomised controlled trial to assess the cardiovascular health effects of a managed aquifer recharge initiative to reduce drinking water salinity in southwest coastal Bangladesh: Study design and rationale. BMJ Open.

[39] Benneyworth L., Gilligan J., Ayers J.C., Goodbred S., George G., Carrico A., Karim M.R., Akter F., Fry D., Donato K. (2016). Drinking water insecurity: Water quality and access in coastal south-western Bangladesh. Int. J. Environ. Heal. Res..

[40] Khan A.E., Scheelbeek P.F.D., Shilpi A.B., Chan Q., Mojumder S.K., Rahman A., Haines A., Vineis P. (2014). Salinity in drinking water and the risk of (pre)eclampsia and gestational hypertension in coastal Bangladesh: A case-control study. PLoS ONE.

[41] Al Nahian M., Ahmed A., Lázár A.N., Hutton C.W., Salehin M., Streatfield P.K. (2018). Drinking water salinity associated health crisis in coastal Bangladesh. Elem. Sci. Anth..

[42] Scheelbeek P.F., Khan A.E., Mojumder S., Elliott P., Vineis P. (2016). Drinking water sodium and elevated blood pressure of healthy pregnant women in salinity-affected coastal areas. Hypertension.

[43] Peters C.N., Baroud H., Hornberger G.M. (2019). Multicriteria decision analysis of drinking water source selection in southwestern Bangladesh. J. Water Resour. Plan. Manag..

[44] WHO (2011). Safe. Drinking Water from Desalination: Guidance on Risk Assessment and Risk Management Procedures to Ensure the Safety of Desalinated Drinking Water.

[45] Pugsley A., Zacharopoulos A., Mondol J.D., Smyth M. (2018). Solar Desalination Potential Around the World. Renewable Energy Powered Desalination Handbook.

[46] Shamsuzzoha M., Rasheduzzaman M., Ghosh R.C. (2018). Building resilience for drinking water shortages through reverse osmosis technology in coastal areas of Bangladesh. Procedia Eng..

[47] Kazakis N. (2018). Delineation of suitable zones for the application of managed aquifer recharge (MAR) in coastal aquifers using quantitative parameters and the analytical hierarchy process. Water.

[48] Tzoraki O., Dokou Z., Christodoulou G., Gaganis P., Karatzas G. (2018). Assessing the efficiency of a coastal Managed Aquifer Recharge (MAR) system in Cyprus. Sci. Total Environ..

[49] BGR (2008). Groundwater and Climate Change: Challenges and Possibilities. https://www.bgr.bund.de/EN/Themen/Wasser/Produkte/Downloads/groundwater_climate_change_pdf.pdf?__blob=publicationFile&v=3.

[50] Rodríguez-Escales P., Canelles A., Sanchez-Vila X., Folch A., Kurtzman D., Rossetto R., Fernández-Escalante E., Lobo-Ferreira J., Sapiano M., San-Sebastián J. (2018). A risk assessment methodology to evaluate the risk failure of managed aquifer recharge in the Mediterranean Basin. Hydrol. Earth Syst. Sci..

